# Relationship Between Onychomycosis and HIV: A Systematic Review

**DOI:** 10.3390/jof12050360

**Published:** 2026-05-13

**Authors:** Samantha Cruz-López, Emiret Analy Albavera-Ramírez, Roberto Arenas, Claudia Erika Fuentes-Venado, Claudia Camelia Calzada-Mendoza, Eunice D. Farfán-García, Juan Castillo-Cruz, Edwin Chávez-Gutiérrez, Erick Martínez-Herrera, Rodolfo Pinto-Almazán

**Affiliations:** 1 Micology Section, Hospital General Dr. Manuel Gea González, Mexico City 14080, Mexico; samanthacrlpz@gmail.com (S.C.-L.); emiret.ar@gmail.com (E.A.A.-R.); rarenas98@hotmail.com (R.A.); 2 Internal Medicine, Hospital General Tacuba ISSSTE, Lago Ontario 36, Tacuba, Mexico City 11410, Mexico; 3 Internal Medicine, Hospital General Regional con Medicina Familiar No. 1 “Lic. Ignacio García Téllez”, Instituto Mexicano del Seguro Social, Plan de Ayala, Cuernavaca 62430, Mexico; 4 Sección de Estudios de Posgrado e Investigación, Escuela Superior de Medicina, Instituto Politécnico Nacional, Plan de San Luis y Díaz Mirón, Mexico City 11340, Mexico; cefvenado@hotmail.com (C.E.F.-V.); cccalzadam@yahoo.com.mx (C.C.C.-M.); efarfang@ipn.mx (E.D.F.-G.); juancast0508@gmail.com (J.C.-C.); 5 Servicio de Medicina Física y Rehabilitación, Hospital General de Zona No. 197, Texcoco 56160, Mexico; 6 Unidad de Investigación, Hospital Regional de Alta Especialidad de Ixtapaluca (HRAEI) Dependiente del IMSS-BIENESTAR, Carretera Federal México-Puebla Km 34.5, Ixtapaluca 56530, Mexico; chz_edwin.bioexp@hotmail.com; 7 Centro de Mezclas Institucional, Hospital Juárez de México, Av. Instituto Politécnico Nacional, Mexico City 07760, Mexico; 8 Fundación Vithas, Grupo Hospitalario Vithas, 28043 Madrid, Spain; 9 Eficiency, Quality, and Costs in Health Services Research Group (EFISALUD), Galicia Sur Health Research Institute (IISGS), Servizo Galego de Saúde-Universidade de Vigo (UVIGO), 36213 Vigo, Spain

**Keywords:** onychomycosis, HIV, *Trichophyton rubrum*, distal lateral subungual onychomycosis

## Abstract

Background/Objectives: Onychomycosis is a fungal nail infection that may present with severe, atypical, or treatment-resistant features in people living with HIV. Despite its clinical importance, evidence regarding its epidemiology, causative agents, and relationship with immune status remains limited. This systematic review aimed to evaluate the association between onychomycosis and HIV, focusing on prevalence, clinical characteristics, etiologic agents, and CD4+ T lymphocyte counts at diagnosis. Methods: A systematic review was conducted following PRISMA guidelines. MEDLINE/PubMed, SciELO, Scopus, and Scilit were searched for studies published between October 2015 and July 2025 in English and Spanish. Eligible studies included case reports, case series, and observational studies involving people with HIV and confirmed onychomycosis. Data extraction was performed independently, and findings were analyzed descriptively. Results: Thirty studies comprising 1296 patients were included; 306 had detailed clinical descriptions. Most cases were reported in the Americas (85.8%) and predominantly involved male patients. CD4+ counts were available in 123 individuals; 52% had <200 cells/µL, including 18 with <50 cells/µL. *Trichophyton rubrum* was the most frequently identified etiologic agent. Conclusions: Onychomycosis in HIV shows etiologic diversity and commonly affects patients with advanced immunosuppression, though it may also occur with partial immune preservation. Prospective standardized studies are needed.

## 1. Introduction

Since its discovery in 1981, human immunodeficiency virus (HIV) infection has become a global public health challenge, with approximately 1.5 million new infections each year despite decades of prevention and treatment efforts. By 2024, 40.8 million people worldwide were living with this infection. Although incidence has declined in regions with effective prevention strategies, HIV persists among vulnerable populations and in areas with limited access to health services [1,2,3].

HIV infection is characterized by progressive immunosuppression due to depletion of CD4+ T lymphocytes (CD4+ T cells) and persistent viral replication. This process predisposes people living with HIV to opportunistic infections, neoplasms, and multisystem clinical manifestations. Acquired immunodeficiency syndrome (AIDS) represents the most advanced stage of HIV infection and is defined by the presence of opportunistic infections, definitional neoplasms, or an absolute CD4+ T lymphocyte count below 200 cells/µL as a consequence of severe immunosuppression. Combination antiretroviral therapy (cART) has transformed the natural history of the infection; however, immune restoration is not complete in 15 to 30% of cases, leaving a persistent risk of cutaneous and systemic infections [3,4,5,6].

Dermatologic manifestations are common in people with HIV, affecting up to 95% of patients. These include infectious and neoplastic conditions, which may be AIDS-defining or present more severely than in immunocompetent individuals. A thorough dermatologic evaluation is essential in HIV care, as it provides early diagnostic clues, reflects immune status, and supports timely therapeutic decisions. Among the broad spectrum of cutaneous manifestations, mycoses are particularly relevant due to their high prevalence in immunocompromised individuals. Fungi such as dermatophytes, yeasts, and non-dermatophytic molds (NDMs) can cause superficial chronic infections that tend to be more extensive, recurrent, or resistant to treatment in people with HIV [7,8,9,10].

Onychomycosis, a fungal infection of the nail unit, accounts for up to 50% of nail disorders in the general population and is reported to be more frequent in adults living with HIV. In Latin American populations, up to 41% of clinically suspected cases of onychomycosis in people with HIV are microbiologically confirmed, with dermatophytes as the predominant etiologic agents [10,11,12].

This infection can affect both fingernails and toenails and is caused by dermatophytes, yeasts, and non-dermatophytic molds. It is characterized by discoloration, thickening, roughness, and onycholysis. Based on clinical features, onychomycosis is classified as distal lateral subungual onychomycosis (DLSO), proximal subungual onychomycosis (PSO), total dystrophic onychomycosis (TDO), white superficial onychomycosis (WSO), endonyx (EO), and mixed pattern onychomycosis (PMO) [10,12,13,14].

The relationship between HIV and onychomycosis reflects interactions among immune status, etiologic agents, and epidemiological factors. Given the heterogeneity and limitations of the available evidence, a structured synthesis is needed to better describe these findings. This systematic review aims to summarize the available literature, focusing on clinical presentation, etiologic agents, and CD4+ T cell count at the time of onychomycosis diagnosis in people living with HIV.

## 2. Materials and Methods

### 2.1. Identification

The review was conducted based on the Preferred Reporting Items for Systematic Reviews and Meta-Analyses (PRISMA) [15]. An advanced search in the databases: Medical Literature Analysis and Retrieval System Online (MEDLINE/PubMed), Scientific Electronic Library Online (SciELO), SCOPUS and Scilit was performed using the terms “onychomycosis” and “HIV, AIDS, Human Immunodeficiency Virus,” seeking patients infected with HIV with onychomycosis confirmed by mycological studies in case reports, case series, clinical trials, and observational studies, published between October 2015 and July 2025, in English and Spanish. A total of 234 articles were found.

### 2.2. Selection and Screening Standards

To ensure data accuracy, 143 duplicate articles across databases were removed. Six articles with non-retrievable abstracts or full texts were also excluded. Exclusion criteria included book chapters, reviews and systematic reviews, studies not focused on onychomycosis, studies addressing only therapy or genetic mutations and studies with incomplete information. Titles, abstracts, and full texts of each potential article were evaluated by two independent reviewers (Cruz-López and Albavera-Ramírez), and inclusion details were resolved by consensus among three reviewers (Arenas, Martínez-Herrera, and Pinto-Almazán).

### 2.3. Data Acquisition

**Figure 1:**
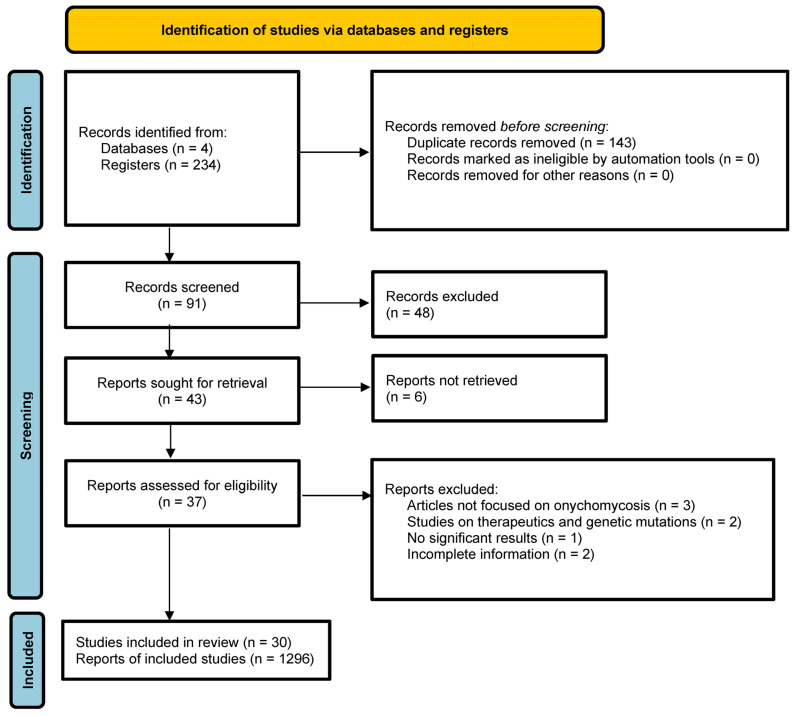
Flowchart of the different phases of the systematic review.

After this process, 85 articles were eligible for assessment. Of these, 48 articles were removed after screening titles and abstracts, leaving 37 for full reading. Finally, 7 were excluded for meeting any exclusion criteria. After screening, 30 articles were included (Figure 1). Data were collected on the following characteristics: total number of diagnosed cases, geographic location, demographic characteristics (sex and age), topography of onychomycosis and number of nails affected (when available), direct examination results (when available), etiologic agents (when determinable), and CD4^+^ T lymphocyte count at the time of infection diagnosis (when reported). Data extraction was performed by two reviewers (Cruz-López and Albavera-Ramírez) using Microsoft Excel, Microsoft Office 2021.

### 2.4. Quality Assessment

To assess the risk of measurement bias in diagnosing onychomycosis, we systematically analyzed the definitions used in the included articles. Identification of fungal species was based on the taxonomies used in the included studies, acknowledging recent taxonomic updates; likewise, the classification of the clinical forms of onychomycosis was maintained according to the criteria proposed by the original authors. When a detailed case description was available, data were documented and compared in Table 1. Conversely, when the percentage of reported cases was the only available datum, the total number of cases (based on the total number of patients included in the study) was calculated and subsequently reported in Table 2. To assess the quality of this study, the PRISMA 2020 checklist [15] was used. No attempt was made to contact the authors of studies with limited case information. This decision was due to time and resource constraints for the review, as well as the low likelihood of obtaining a response within the study timeframe.

## 3. Results

Thirty articles met the inclusion criteria: case reports *(n* = 7) [16,17,18,19,20,21,22], one case series (*n* = 1) [23], and observational studies (*n* = 22) [24,25,26,27,28,29,30,31,32,33,34,35,36,37,38,39,40,41,42,43,44,45], comprising 1296 patients (Table 2). The Americas accounted for the highest number of cases, with 1112 cases reported to date (85.8%), with the United States contributing the largest proportion [64.2% (*n* = 833)], followed by Mexico [6.4% (*n* = 83)] and Brazil [4.1% (*n* = 54)]. In Asia, 80 cases (6.1%) were reported in India; in Europe, 76 cases (5.8%) were reported from Greece; and in Africa, 22 cases (1.69%) were reported from Nigeria and South Africa. Eurasia had the fewest diagnosed cases, with 20 patients (1.54%) in Turkey.

Seven case reports [16,17,18,19,20,21,22], one case series [23], and ten observational studies [24,26,27,29,32,35,37,39,40,42] of the total number of articles included, reported a detailed description of diagnosed patients, comprising a total of 306 individuals. Of these, 69.6% (*n* = 213) were male. CD4 T-lymphocyte count was reported heterogeneously across the included studies and was identified in 123 patients (40.2%). Individual-level data were extracted when available. Any quantitative CD4+ T-cell count expressed as an absolute value, mean, median, range, or clinical category was considered as reported. In studies that reported CD4+ T-lymphocyte count at the group level, the reported value was used to classify patients into immunological strata. For patients with multiple measurements, the value corresponding to the time of diagnosis was used. In this regard, 18 patients (5.8%) had <50 cells/µL, 46 (15%) had counts between 50–199 cells/µL, 41 patients were within the 200–499 cells/µL range, and 18 (5.8%) had a count of 500 or more cells/µL.

Regarding the etiological agent of onychomycosis, it was individually or specifically identified in 252 patients; among these, *Trichophyton rubrum* was most frequently isolated [43.6% (*n* = 110)], followed by *Candida* spp. [11.9% (*n* = 30)] and *Trichophyton* spp. [5.5% (*n* = 14)]. In eight cases (3.1%), *Candida* spp. and *Trichophyton* spp. coexisted, and in the same proportion (3.1%), *Candida parapsilosis* was isolated. *Candida albicans* was identified in 7 cases (2.7%), and *Trichophyton mentagrophytes* and *Trichophyton interdigitale* in 4 cases each (1.5%). *Kodamaea ohmeri* and *Trichosporon asahii* were isolated in three patients each (1.1%). *Epidermophyton floccosum*, *Trichosporon mucoides, Candida haemulonii*, and *Cryptococcus laurentii* were identified in two cases each (0.7%), and *Aspergillus sydowii*, *Candida tropicalis*, *Microsporum gypseum*, *Penicillium brasilianum*, *Trichophyton tonsurans*, and *Stephanodiscus ciferrii* in one case each (0.3%). In 33 patients (13.09%), the etiological agent was not reported, and in 13 (5.1%), cultures showed no growth. In one study that included the remaining 54 patients with onychomycosis and HIV [26], the etiological agents isolated for each individual weren’t specified; however, they are relevant to this review; It was reported that 25 patients were positive for a single pathogen, 5 for two agents, 2 patients had three microorganisms isolated, and 1 patient had no isolate obtained. Among these, *Candida parapsilosis* was reported on 12 occasions; *Candida* spp., *Candida guilliermondii*, *Rhodotorula mucilaginosa*, *Mucor* spp., and *Penicillium* spp. in three occasions; *Trichosporon mucoides* and *Fusarium oxysporum* in two occasions; and *Candida albicans, Aspergillus* spp., *Scytalidium* spp., *Geotrichum capitatum*, *Hormonema dematioides*, *Cryptococcus uniguttulatus*, *Monilia sitophila*, *Fusarium* spp., and *Aspergillus niger* in one occasion each.

Among the remaining studies, eight case reports and one case series [16,17,18,19,20,21,22,23] provided explicit information on 14 patients. The mean age at presentation was 45.2 years (range, 32–69 years). Reported comorbidities included mycoses in 35.7% (*n* = 5), viral blistering diseases in 21.4% (*n* = 3), and other conditions in 7.1% (*n* = 1); similarly, 7.1% (*n* = 1) was reported as healthy, and in 42.8% of patients (*n* = 6) no information regarding medical history was provided. The mean duration of onychomycosis was reported in 35.7% (*n* = 5), with a maximum of 1.6 years. A total of 54 affected nails were recorded. Toenail involvement was predominant, accounting for 53% (*n* = 29), whereas fingernails represented 46.2% (*n* = 25). In one case, fingernails were reported as involved without specifying the number, and in five cases involving toenails the same situation occurred. Regarding the clinical presentation of onychomycosis, 20.3% (*n* = 11) presented PSO, 18.5% (n = 10) TDO, while WSO and DLSO each accounted for 1.8% (*n* = 1). In the six patients for whom the number of affected nails was not obtained, dermatophytoma was reported: five with WSO and one with DLSO. As diagnostic methods, direct potassium hydroxide examination and culture were used together in 78.5% (*n* = 11) of patients, and biopsy was performed in 14% (*n* = 2); in 2 cases (14.2%), molecular biology techniques were added to these methods.

Regarding antiretroviral therapy for HIV infection, 14.2% of patients were receiving a regimen, 28.5% were not undergoing treatment, and in 50% (*n* = 7) this information could not be obtained. Concerning onychomycosis treatment, it could be clearly analyzed in 21.4% (*n* = 3) of patients, as this subgroup did not present concomitant mycoses that modified the therapeutic regimen. Oral itraconazole monotherapy was used in 7.1% (*n* = 1), oral fluconazole monotherapy in 7.1% (*n* = 1), and the combination of oral terbinafine and topical ketoconazole in 7.1% (*n* = 1). Regarding therapeutic response, no clinical outcome was reported in 28.5% of cases (*n* = 4) after antifungal treatment, as this information was not mentioned in the studies. Among those in which outcomes were specified, resolution of onychomycosis was reported in 7.1% (*n* = 1), clinical improvement in 7.1% (*n* = 3), and good initial clinical response in 7.1% (*n* = 1).

## 4. Discussion

The association between onychomycosis and HIV infection has been described with more aggressive clinical features, including greater involvement of the nail plate surface, a higher number of affected nails, and refractoriness to treatment [23,35]. Immunocompetent patients with onychomycosis typically present involvement of one or both feet and a single hand; however, when nail involvement affects all four extremities, an underlying immunocompromised state should be suspected [16]. In this context, onychomycosis may be the first and only presenting sign of HIV infection, representing a potential marker for timely diagnosis and treatment [41].

Onychomycosis may occur as an isolated condition or as part of generalized dermatophytosis; it is a chronic fungal infection affecting multiple body sites and differs from disseminated dermatophytosis in that the dermatophyte does not invade the dermis nor spread to subcutaneous tissue or lymph nodes. In individuals infected with HIV, the term onycho-mucocutaneous syndrome has likewise been coined to indicate the increased likelihood of involvement of the skin, mucosa, and nails. These conditions have been reported in patients with CD4+ T-lymphocyte counts below 200 cells/µL [17,20].

The incidence of onychomycosis in immunocompromised patients is increasing; in people living with HIV, this clinical entity represents one of the early manifestations of infection, with a prevalence ranging from 15% to 40%, approximately four times higher than in the general population [18,22,32,35,41]. Our results suggest a predominance of cases in the Americas [85.8% (*n* = 112)], particularly in the United States [64.2% (*n* = 833)], followed by Mexico [6.4% (*n* = 83)] and Brazil [4.1% (*n* = 54)], which likely reflects greater diagnostic and reporting capacity. However, this distribution should be interpreted with caution, as it likely reflects differences in diagnostic capacity, surveillance systems, and scientific output, rather than a true global epidemiology. In several countries in the Americas, there is greater access to mycological diagnostic tools, facilitating the identification of typical and atypical presentations of onychomycosis in people with HIV. Conversely, regions with a high prevalence of HIV infection, such as sub-Saharan Africa and parts of Asia, may be underrepresented due to limited diagnostic infrastructure, reduced access to specialized care, and a lack of reporting in indexed literature. Furthermore, publication bias may contribute to this pattern, as studies from the Americas are more frequently indexed in international databases. Therefore, the apparent predominance in this region likely represents a combination of improved detection, more thorough clinical follow-up, and a reporting mechanism [1,2]. Likewise, a predominance of male sex was identified [59.7% (*n* = 169)], a finding consistent with other series. However, some authors have reported greater susceptibility to fungal infections, particularly onychomycosis, in women, suggesting that sociocultural, hormonal, and healthcare access factors may influence this variability [28,31]. Regarding ethnic origin or ancestry, Bender et al. reported in one series a higher coexistence of treated infections among African American patients [31]. When attempting to analyze age and number of affected nails in patients from these articles, we found a significant lack and heterogeneity of detailed information regarding these variables; therefore, a comprehensive evaluation was not possible.

Cellular immunosuppression plays a crucial role in the development of HIV-associated onychomycosis. Several studies have shown that dysregulation of regulatory T lymphocytes (CD4+ CD25+ FoxP3+) may favor persistence of nail infection by suppressing cytotoxic T-lymphocyte responses and attenuating protective inflammation. Additionally, an increase in regulatory T lymphocytes has been described both in patients with onychomycosis and in individuals with HIV, associated with viral load; this alteration tends to normalize with effective antiretroviral therapy but persists in patients without therapeutic response. Onychomycosis may manifest in patients with varying levels of immunosuppression, driven by this mechanism; studies have demonstrated that CD4+ T-lymphocyte counts below 200–450 cells/µL are associated with a greater frequency and severity of nail disease [23,24,35,37]. In this review, CD4 T-lymphocyte count was available in 123 of 306 patients, of whom 64 (52.0%) had values <200 cells/µL, including 18 patients with CD4 < 50 cells/µL, evidencing a predominance of advanced immunosuppression at diagnosis and reinforcing the hypothesis that onychomycosis may act as a visible clinical marker of the degree of immunodeficiency [35]. Nevertheless, 59 patients had counts ≥200 cells/µL, including 18 with CD4 ≥ 500 cells/µL, indicating that onychomycosis may also occur in people living with HIV with partial or preserved immunity. The heterogeneity in CD4+ T-cells reporting and the absence of this information in a relevant proportion of studies limit quantitative synthesis and highlight the need to standardize immunological assessment in future research.

Regarding etiology, it is important to emphasize that in this review, the scientific names of etiological agents were used as reported in the reviewed articles; however, according to the 2017 nomenclature, some have changed due to polyphasic studies (macro and microscopies, biochemical analysis, secondary metabolites, and molecular biology) [46]. Various fungi were reported in the reviewed articles, including dermatophytes, yeasts, and non-dermatophyte molds [23,26]. Our findings confirm that *Trichophyton rubrum* [43.6% (*n* = 110)] is the main etiological agent of onychomycosis in people with HIV, followed by other species of the genus *Candida* spp. [11.9% (*n* = 30)] and the genus *Trichophyton* spp. [5.5% (*n* = 14)], consistent with previous reports [17,23]. However, considerable diversity of emerging yeasts and NDM was observed, such as *Kodamaea ohmeri*, *Trichosporon* spp., *Aspergillus sydowii*, and *Penicillium brasilianum*; this phenomenon has been more frequently reported in patients with HIV, in whom non-dermatophyte molds may account for up to four times more cases than in the general population [35]. Moreover, the isolation of emerging species is highly significant, as some exhibit antifungal resistance attributed to their high capacity for biofilm formation and adhesion to keratin substrates, complicating treatment and favoring persistence of infection. In particular, resistance to azoles and terbinafine has been documented in *Candida* species, secondary to prolonged fluconazole use, which in this context may be related to prophylaxis and treatment of opportunistic infections in people with HIV [28,29]. An example is the report of the *Candida haemulonii* complex, which highlights the importance of proper microorganism identification, as it is phylogenetically related to species such as *Candidozyma auris* formerly known as *Candida auris*; therefore, molecular rather than solely phenotypic tools are necessary for accurate categorization, as the treatment is different for both [47].

On the other hand, it should be clarified that the reported cases of Trichosporon spp. were carried out with molecular biology (VITEK and VITEK-2) [26,29] since it has been reported that this microorganism shares antigenic reactivity with *Cryptococcus neoformans* when the diagnosis of the latter is made by lateral flow immunochromatographic assays through detection of cell-wall glucuronoxylomannan (GXM), thus highlighting the importance of carrying out molecular biology studies in order to avoid this bias [48].

From a clinical standpoint, total dystrophic onychomycosis, which represented the second most frequent presentation [18.5% (*n* = 10)], together with distal lateral subungual onychomycosis, are the main patterns observed in people with HIV. However, proximal subungual onychomycosis and proximal white onychomycosis, considered uncommon in immunocompetent individuals, have been regarded as more characteristic and highly suggestive forms of HIV infection. PSO is characterized by proximal leukonychia in the lunular region; it represented the most frequent type in our study [22.2% (*n* = 12)] [21,24,28,35,37,40,41]. Likewise, the presence of dermatophytoma should be highlighted, as it is associated with poor response to conventional treatment [23].

Regarding diagnosis, our results reinforce the need for a comprehensive mycological approach in patients with HIV, as isolated clinical evaluation is insufficient. The combined use of direct potassium hydroxide examination and mycological culture was the most frequently employed diagnostic method [78.5% (*n* = 11)]. In some cases, nail biopsy and molecular biology techniques were used [14% (*n* = 2)], consistent with current recommendations to improve diagnostic accuracy, especially in cases involving non-dermatophyte agents or mixed infections.

The introduction of cART has significantly modified the natural history of onychomycosis in people with HIV, reducing its incidence and, in some cases, favoring improvement in the Onychomycosis Severity Index (OSI) score or spontaneous resolution without the need for systemic antifungals. This phenomenon may be explained by immune reconstitution, increased CD4+ count, accelerated nail growth in these patients, and partial normalization of antifungal response, underscoring the central role of virological control in the comprehensive management of HIV-associated onychomycosis [24,25,35]. On the other hand, adverse effects of this therapy may impact the nail plate and should be considered in the differential diagnosis of onychomycosis in patients receiving these regimens; longitudinal melanonychia, bluish discoloration of the lunula, and paronychia have been observed in patients receiving cART with zidovudine, while protease inhibitors may cause pyogenic granulomas [42,44]. These findings could not be effectively corroborated in our data, as only 14.2% of patients were receiving treatment, 28.5% were untreated, and in 50% (*n* = 7) this information was unavailable.

Although treatment analysis was not included in the primary objective, it was observed that many articles did not report the therapeutic regimen used or its outcome. It should be noted that no standardized clinical guidelines currently exist for the management of onychomycosis in people living with HIV. Oral terbinafine remains the first-line treatment in the general population, with cure rates close to 60% and relapse rates ranging from 20% to 50% [32]; however, its efficacy may be limited by resistant species, the emergence of new pathogenic fungal strains, drug interactions with antiretroviral therapy, and the risk of hepatotoxicity. Consequently, innovative therapies, such as the topical application of Vicks VapoRub, have been proposed. However, one study demonstrated significant effectiveness; further studies are required to compare this option with placebo or standard treatment and to understand its efficacy better [28,32]. In our review, therapeutic information was limited and heterogeneous; nevertheless, in cases where treatment was documented, oral itraconazole and oral fluconazole monotherapy, and the combination of oral terbinafine with topical ketoconazole were each used in 7.1% of patients [17,20,22]. Partial improvement was primarily observed, reflecting the inherent challenges in managing this condition in immunocompromised patients and the lack of treatment reporting in these cases [17,19,20,21,22].

## 5. Limitations

During this review, a limitation in the epidemiological analysis was found due to a considerable lack of data, particularly regarding CD4+ T cell count, age, sex, number of affected nails, and antiretroviral treatment status, which prevented consistent comparisons. This is a descriptive study; therefore, we cannot establish direct associations but rather report the findings available in the literature, highlighting the spectrum of clinical presentations, etiological agents, and diagnostic considerations in this population. Specifically, the limitation of the CD4 T lymphocyte count highlights the importance of systematically including the CD4+ T lymphocyte count in future studies/reports, as it would enable a more accurate correlation between the clinical manifestations of onychomycosis and the patient’s immune status. Likewise, information on antifungal treatment and its outcomes was incomplete, hindering the evaluation of therapeutic efficacy. This underscores the need for greater precision and standardization in reporting information in these patients to enable more accurate assessments in future studies.

## 6. Conclusions

This systematic review confirms that onychomycosis is a clinical condition reported in people living with HIV, although its frequency and overall clinical impact cannot be determined from the predominantly descriptive and case-based literature. This gap limits understanding of the disease burden, the natural history of onychomycosis in HIV, and appropriate therapeutic strategies.

Our findings indicate that onychomycosis in people with HIV involves a wider range of causative agents and atypical clinical forms. Therapeutic data are especially limited, as most studies do not provide details on antifungal regimens, treatment duration, or clinical and mycological outcomes. This lack of evidence hinders the development of specific treatment recommendations and underscores the need for prospective studies and clinical trials to evaluate the efficacy, safety, and cost-effectiveness of antifungal options in this population.

This review highlights the urgent need for more comprehensive reporting and description of onychomycosis cases in people with HIV. Strengthening the evidence base will improve diagnosis and treatment and may establish this condition as an early clinical marker of immune status and infection control, supporting timely and comprehensive care for this vulnerable population.

## Figures and Tables

**Table 1:** Clinical reports of patients with HIV and onychomycosis.

Country	Age	Sex	Onset	Topography		Presentation of Pathology	Diagnostic Method	Etiological Agent	Comorbidities	CD4 Count at Diagnosis	Viral Load		Antiretroviral Treatment		Onychomycosis Treatment	Outcome	Ref.
				Hands (Number of Fingers Affected)	Foot (Number of Fingers Affected)						Before Diagnosis	After Diagnosis	Before Diagnosis	After Diagnosis			
Argentina	48	M	N/A	10	10	TDO	KOH and culture	*Trichophyton rubrum*	*Cryptococcus* *pneumoniae*	1680 cells/µL	N/A	N/A	N/A	N/A	Liposomal amphotericin B 3–4 mg/kg/day and fluconazole 1200 mg/day.	N/A	[16]
India	50	M	1 year	1	0	PSO	KOH and culture	*Trichophyton mentagrophytes*	Extragenital molluscum contagiosum and chronic pseudomembranous oral candidiasis	57 cells/µL	N/A	N/A	None	Tenofovir + Lamivudina + Efavirenz	Topic Luliconazole and intravenous fluconazole.	N/A	[17]
India	32	M	1 month	1	0	PSO	KOH and culture	*Trichophyton mentagrophytes*	Herpes genitalis	N/A	N/A	N/A	None	Yes	Oral itraconazole	Significant improvement	
India	33	M	N/A	1	0	PSO	KOH, culture and molecular identification	*Aspergillus* *sydowii*	Ringworm of the body	239 cells/µL	N/A	N/A	Tenofovir + Lamivudina + Efavirenz	Tenofovir + Lamivudina + Efavirenz	N/A	N/A	[18]
Spain	48	M	1 month	0	1	PSO	KOH and culture	*Trichophyton rubrum*	None	40 cells/µL	N/A	17,510 copies/µL	None	Yes	Antifungal therapy	Resolution of onychomycosis.	[19]
USA	62	F	N/A	1	0	TDO	Biopsy with PAS. KOH and culture.	*Trichophyton rubrum*	Untreated hepatitis C, severe chronic obstructive pulmonary disease, *tinea capitis*, *tinea corporis.*	135 cells/µL	N/A	N/A	Noncompliance	N/A	Fluconazole 200 mg daily,	Clinical improvement	[20]
Argentina	34	M	N/A	0	8	PSO	KOH and culture	*Microsporum gypseum.*	Subacute disseminated histoplasmosis, acalculous cholecystitis, deep vein thrombosis, HSV, *tinea corporis*	16 cells/µL	182.000 copies/mL	N/A	None	Emtricitabine + tenofovir + atazanavir	Terbinafine 250 mg daily	Good initial clinical response	[21]
USA	69	M	Few weeks	1	0	DLSO with dermatophytoma	Biopsy with PAS, culture and DNA sequence analysis.	*Penicillium brasilianum.*	Coronary artery disease	1105 cells/μL	N/A	32 copies /mL	Dolutegravir + lamivudine + abacavir.	Dolutegravir + lamivudine + abacavir.	terbinafine 250 mg daily and topical ketoconazole 2% cream.	Clinical improvement	[22]
Colombia	33	M	73 months	Unspecified number of fingers		DLSO with dermatophytoma	KOH and culture	*Epydermophyton floccosum*	N/A	282 cells/µL	N/A	N/A	N/A	N/A	N/A	N/A	[23]
	40	M			Unspecified number of fingers	WSO with dermatophytoma		*Trichophyton rubrum*		78 cells/µL							
	42	M				WSO with dermatophytoma		*Trichophyton tonsurans*		25 cells/µL							
	44	M				WSO with dermatophytoma		Negative culture		20 cells/µL							
	49	M				WSO with dermatophytoma		*Trichophyton mentagrophytes*		9 cells/µL							
	50	F				WSO with dermatophytoma		*Trichophyton mentagrophytes*		107 cells/µL							

M (male), F (female). The shaded area represents the finger with the affected nail. N/A (Not available). TDO (Total dystrophic onychomycosis), PSO (Proximal subungual onychomycosis), DLSO (Distal lateral subungual onychomycosis), WSO (White superficial onychomycosis), KOH (Potassium hydroxide).

**Table 2:** Global distribution of reported cases with diagnosis of onychomycosis in patients with HIV.

Continent	Country/Region	No. of Cases per Article	No. of Cases per Country	No. of Cases per Continent	Reference
**America**	**Mexico**	16	83	1102	[24]
		13			[25]
		54			[26]
	**Peru**	51	51		[27]
	**Brazil**	3	54		[28]
		44			[29]
		7			[30]
	**Maryland**	248	833		[31]
	**Washigton**	20			[32]
	**United States**	556			[33]
	**Texas**	9			[34]
	**Ecuador**	42	42		[35]
	**Cuba**	39	39		[36]
**Euroasia**	**Turkey**	5	20	20	[37]
	**Turkey**	15			[38]
**Europe**	**Greece**	75	75	75	[39]
**Asia**	**India**	2	77	77	[40]
	**Shillong, India**	38			[41]
	**Bangalore, India**	3			[42]
	**India**	34			[43]
**Africa**	**Nigeria**	17	17	22	[44]
	**South Africa**	5	5		[45]

## Data Availability

Data extracted are available within the manuscript or from the corresponding author upon reasonable request.
